# Postoperative efficacy of transurethral resection of the prostate (TURP) in treating benign prostatic hyperplasia combined with detrusor underactivity

**DOI:** 10.1097/MD.0000000000046527

**Published:** 2025-12-12

**Authors:** Yuhong Xin, Dai Li, Shuzhen Lin, Riqiang Gan, Lijuan Li, Zhenhua Zhao, Aili Zhao, Chao Yang, Bingkun Li

**Affiliations:** a Department of Urology, People’s Hospital of Jiangmen, Jiangmen, China; b Department of Anesthesiology, People’s Hospital of Jiangmen, Jiangmen, China; c Department of Urology, Zhujiang Hospital, Southern Medical University, Guangzhou, China.

**Keywords:** benign prostatic hyperplasia, bladder contractility index, detrusor underactivity, transurethral resection of the prostate, urodynamic examination

## Abstract

This study investigated the postoperative efficacy of transurethral resection of the prostate (TURP) in patients with benign prostatic hyperplasia (BPH) and detrusor underactivity (DU). A total of 163 patients with BPH who underwent TURP at our hospital were analyzed. All the patients were diagnosed with DU via preoperative urodynamic tests. Based on the Schaefer nomogram assessment of detrusor contractility, patients were categorized into 3 groups: very weak (VW) (n = 30), weak minus (W−) (n = 46), and weak plus (W+) (n = 87). Postoperative outcomes, including International Prostate Symptom Scores (IPSS), quality of life (QOL) scores, maximum free urinary flow rate (fQmax), voided volume (VV), and post-void residual volume (PVR), were compared with preoperative values 2 weeks after catheter removal. Before surgery, there were no significant differences in IPSS, QOL, fQmax, VV, and PVR between the groups. Postoperatively, improvements were observed in all variables except for PVR in the VW and W− groups. Significant improvements in IPSS, QOL, and fQmax were noted compared to the preoperative values, but VV and PVR changes were not significant. Patients with normal detrusor strength showed greater improvements. TURP effectively enhanced the urinary flow and subjective urinary symptoms in most patients with BPH and DU. Effectiveness varies with detrusor muscle strength, and patients closer to normal strength experience better outcomes. If a ≥5 mL/s increase in fQmax post-TURP was deemed effective, the success rates were 50.0%, 69.6%, and 81.6% for the Valsalva voiding VW, W−, and W+ groups, respectively, with an overall rate of 72.4%. TURP is less effective when the bladder contractility index is <40 to 42.

## 1. Introduction

Benign prostatic hyperplasia (BPH) is one of the most common causes of lower urinary tract symptoms in middle-aged and elderly men. BPH symptoms include voiding, storage, and post-voiding symptoms, with bladder outlet obstruction (BOO) being the initial factor, manifesting mainly as voiding difficulties and a weak urinary stream due to mechanical obstruction from hyperplasia of prostate tissue compressing the passage between the bladder neck and external urethral sphincter, coupled with dynamic obstruction due to smooth muscle tension.^[1]^ Early reduction of bladder outlet resistance through medication or surgery can effectively improve voiding and protect detrusor muscle function; however, continued high load on the detrusor muscle can lead to detrusor underactivity (DU), making clinical treatment significantly challenging once it progresses to DU.^[2]^ In patients with early BOO, timely intervention can protect bladder detrusor function; however, with continued high-load conditions, DU can develop, where clinical treatment becomes considerably more difficult. There is a lack of consensus on surgical treatments for such cases, especially among those with severe DU, as most doctors believe that these patients do not have significantly improved voiding function postsurgery. As a result, more conservative treatments are often chosen to avoid disputes, although several studies have indicated that these patients can still benefit from surgery.^[3]^ Surgery not only maximizes bladder function preservation, but may also provide an opportunity for bladder function recovery, making surgical intervention advisable.^[4]^ However, previous studies have not clarified whether the degree of DU should guide surgical decision-making. Our hospital conducted transurethral resection of the prostate in these patients with informed consent and found generally acceptable outcomes. This is a detailed description below.

## 2. Materials and methods

1. A review was conducted on BPH patients diagnosed between February 2022 and July 2024 at our hospital, all of whom exhibited symptoms of voiding obstruction or even urinary retention. All patients completed the prostatic specific antigen tests, prostate ultrasound/computed tomography (CT)/magnetic resonance imaging, International Prostate Symptom Scores (IPSS), quality of life (QOL) scores, and urodynamic examinations. Patients with elevated prostatic specific antigen levels first underwent a prostate biopsy to exclude prostate cancer. For patients with high urinary retention (post-void residual urine > 600 mL), indwelling catheters were used for 2 weeks before the urodynamic examination. The exclusion criteria included patients suspected of having neurogenic bladder based on findings, such as those with diabetes, cerebral infarction, Parkinson disease, and previous pelvic surgery history. All enrolled patients had no surgical contraindications and provided informed consent before surgery by qualified and experienced physicians. The surgical procedures were all performed successfully in all cases. The second visit occurred 2 weeks after catheter removal for repeat IPSS, QOL scoring, and reevaluation of the urinary flow rate and post-void residual volume (PVR). All procedures performed in studies involving human participants were in accordance with the ethical standards of the institutional and/or national research committee and with the 1964 Helsinki Declaration and its later amendments or comparable ethical standards. This study has been approved by the Ethics Committee of Jiangmen People’s Hospital.
2. Urodynamic examination qualified personnel were used to ensure accuracy and objectivity using the MMS urodynamic analyzer imported from the Netherlands. During the procedure, International Continence Society standards were followed to determine patients with DU by analyzing the maximum urinary flow rate during the voiding phase and the corresponding detrusor pressure (Pdet.Qmax). The bladder contractility index (BCI) was calculated using the following formula: BCI = Pdet.Qmax + 5Qmax. DU patients were screened based on the Schaefer nomogram, identifying 163 DU cases, and further divided based on DU’s degree into very weak (VW), W−, and W+ groups, with maximum free urinary flow rate (fQmax), voided volume (VV), and PVR recorded after catheter removal at 2-week follow-up.
3. Transurethral resection of the prostate: To ensure procedural fairness and stability, all procedures were performed by 2 qualified and experienced prostate surgeons using transurethral resection of the prostate techniques, with catheters removed 5 to 7 days after surgery.
4. Statistical analysis: SPSS software (version 13.0) was used for statistical analyses, including parameters such as age, IPSS score, QOL score, prostate volume, fQmax, VV, and PVR. Paired-sample *t* tests were used to analyze paired measurements while ensuring homogeneity of variance. Statistical significance was set at *P* < .05.

## 3. Results

**Figure 1.**
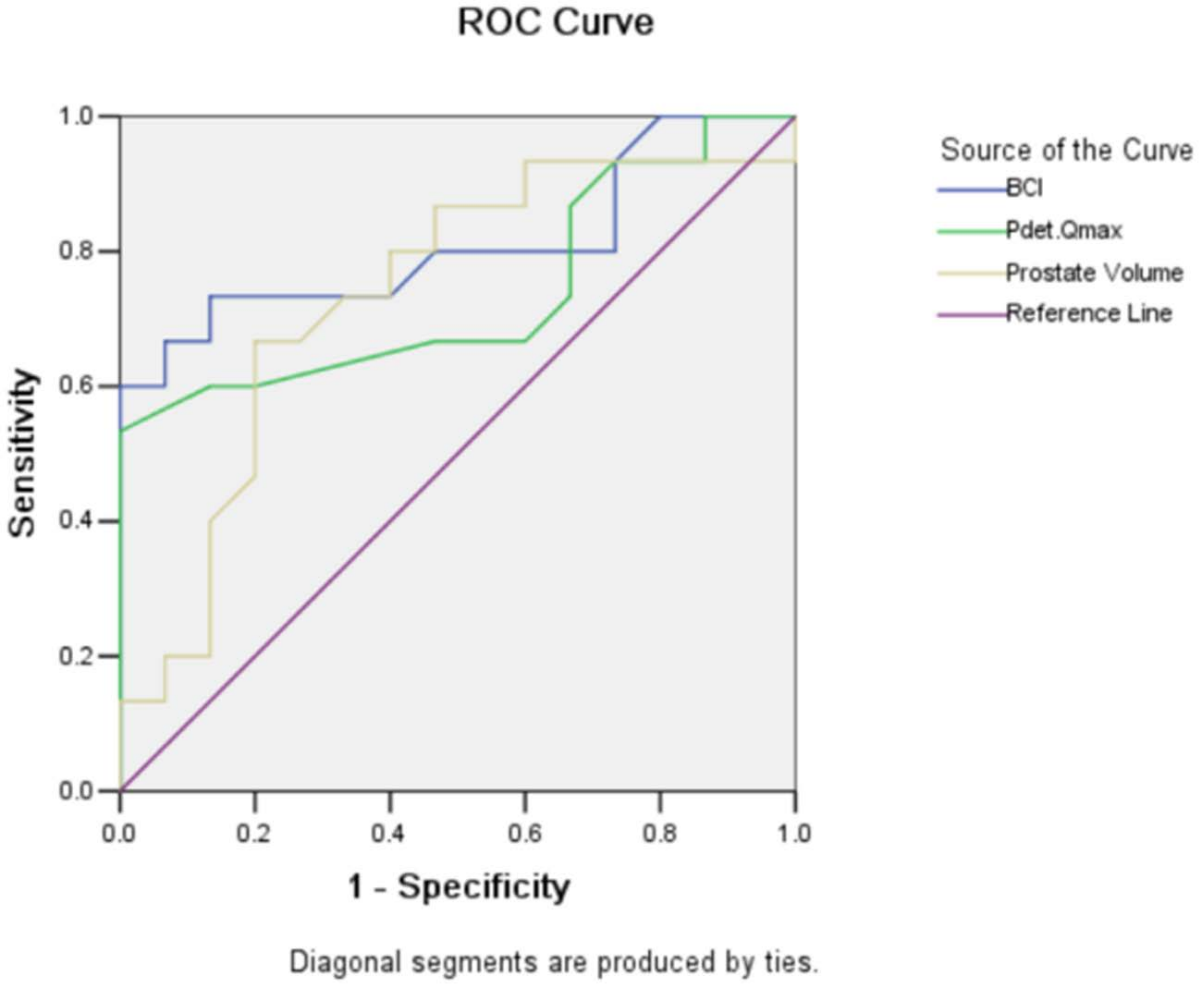
The area under ROC curve of preoperative prarameters.

1. A total of 163 patients, aged 49 to 95 with an average age of 71.9 ± 8.0, showed no significant preoperative differences in age, prostate volume, IPSS, QOL, fQmax, VV, or PVR (*P* < .05) among the groups. See Table 1.
2. Significant differences were observed pre- and postoperatively in IPSS, QOL, fQmax, and VV across all groups (*P* < .05), whereas PVR showed no significant difference (*P* > .05). See Table 2.
3. Comparison of postoperative IPSS scores, QOL scores, fQmax, VV, and PVR differences among different groups is shown in Table 3.
4. An fQmax difference of ≥5 mL/s was defined as treatment effectiveness, and the treatment effectiveness of each group was graded. For the most controversial VW group patients, the sensitivity and specificity of treatment effectiveness were defined using receiver operating characteristic curve analysis to identify which parameters were positively correlated with treatment effectiveness. The area under the ROC curve of each parameter is shown in Table 4. The ROC curve of meaningful parameters is shown in Figure 1, and the sensitivity and specificity corresponding to treatment effectiveness defined by each parameter are shown in Table 5.

**Table 1:** The contrast of preoperative paremeters.

Groups	Case number*	Age (yr)	Prostate volume (mL)	IPSS	QOL	fQmax (mL/s)	VV (mL)	PVR (mL)
VW	30 (12)	73.1 ± 7.7	57.9 ± 22.7	26.7 ± 2.8	4.9 ± 0.5	2.2 ± 3.4	46.3 ± 78.0	243.3 ± 252.9
W−	46 (21)	70.7 ± 8.6	59.2 ± 24.5	26.4 ± 2.7	4.9 ± 0.4	3.0 ± 4.2	67.2 ± 94.7	113.8 ± 179.2
W+	87 (41)	72.2 ± 7.7	68.0 ± 27.5	26.3 ± 3.7	4.9 ± 0.6	3.3 ± 4.3	53.1 ± 72.6	109.8 ± 139.3
*F*		0.968	2.643	0.186	0.043	0.763	0.718	2.943
*P*		.382	.074	.831	.958	.468	.489	.059

fQmax = maximum free urinary flow rate, IPSS = International Prostate Symptom Scores, PVR = post-void residual volume, QOL = quality of life, VV = voided volume, VW = very weak, W− = weak minus, W+ = weak plus.

* The data of residual urine is the number of cases after excluding the patients with urinary retention.

**Table 2:** The contrast of pre- and postoperative paremeters.

Parameters	VW				W−				W+			
	Preoperative	Postoperative	*t*	*P*	Preoperative	Postoperative	*t*	*P*	Preoperative	Postoperative	*t*	*P*
IPSS	26.7 ± 2.8	13.1 ± 6.0	15.021	.000	26.4 ± 2.7	11.3 ± 5.3	17.045	.000	26.3 ± 3.7	8.6 ± 4.3	31.440	.000
QOL	4.9 ± 0.5	3.0 ± 1.0	10.143	.000	4.9 ± 0.4	2.3 ± 1.2	13.159	.000	4.9 ± 0.6	1.7 ± 1.2	22.587	.000
fQmax (mL/s)	2.2 ± 3.4	8.0 ± 4.9	7.937	.000	3.0 ± 4.2	12.3 ± 7.2	9.361	.000	3.3 ± 4.3	15.9 ± 7.5	15.169	.000
VV (mL)	46.3 ± 78.0	119.3 ± 89.0	4.714	.000	67.2 ± 94.7	171.3 ± 96.3	5.467	.000	53.1 ± 72.6	152.4 ± 76.3	10.275	.000
PVR (mL)*	243.3 ± 252.9	114.2 ± 107.4	2.000	.071	113.8 ± 179.2	84.3 ± 117.0	1.082	.292	109.8 ± 139.3	61.8 ± 72.1	2.173	.036

fQmax = maximum free urinary flow rate, IPSS = International Prostate Symptom Scores, PVR = post-void residual volume, QOL = quality of life, VV = voided volume, VW = very weak, W− = weak minus, W+ = weak plus.

* Comparison of residual urine volume: 12 cases in VW group, 21 cases in W− group, and 41 cases in W+ group.

**Table 3:** Comparison of differences in various indicators after surgery among different groups.

Groups	Case number	Difference of IPSS	Difference of QOL	Difference of fQmax (mL/s)	Difference of VV (mL)	Difference of PVR (mL)
VW	30 (12)	13.6 ± 5.0	1.9 ± 1.0	5.8 ± 4.0	73.0 ± 84.8	129.2 ± 223.7
W−	46 (21)	15.1 ± 6.0	2.6 ± 1.4^**^	9.3 ± 6.8^**^	104.1 ± 129.2	29.5 ± 125.0
W+	87 (41)	17.7 ± 5.2*	3.3 ± 1.4*	12.6 ± 7.7*	99.3 ± 90.2	44.9 ± 132.3
*F*		7.657	13.938	11.518	0.957	1.903
*P*		.001	.000	.000	.386	.157

fQmax = maximum free urinary flow rate, PVR = post-void residual volume, QOL = quality of life, VV = voided volume, VW = very weak, W− = weak minus, W+ = weak plus.

The *Q* test results for comparing between each 2 groups show that: * W+ group is compared with W− group and VW group respectively, with *P* < .05. ** The comparison between the W− group and the VW group showed *P* < .05.

**Table 4:** The area under ROC curve of preoperative prarameters.

Parameters	Area under ROC curve	*P* value	95% confidence interval
BCI	0.809	.004	0.644–0.974
Urination period Qmax	0.704	.056	0.513–0.896
Pdet.Qmax	0.738	.026	0.550–0.925
Preoperative fQmax	0.551	.633	0.341–0.762
Preoperative VV	0.524	.820	0.312–0.737
Preoperative IPSS	0.291	.051	0.096–0.486
Preoperative QOL	0.476	.820	0.264–0.687
Prostate volume	0.733	.029	0.546–0.920

BCI = bladder contractility index, fQmax = maximum free urinary flow rate, Pdet.Qmax = maximum urinary flow rate during the voiding phase and the corresponding detrusor pressure, QOL = quality of life, VV = voided volume.

**Table 5:** The sensitivity and specificity of partial preoperative prarameters.

Parameters	Value	Sensitivity	1-specificity
BCI	37.25	0.733	0.200
	39.25	0.733	0.133
	41.25	0.667	0.133
	42.25	0.667	0.067
Pdet.Qmax	31.00	0.600	0.200
	33.50	0.600	0.133
	36.50	0.533	0.000
	39.00	0.333	0.000
Prostate volume	54.50	0.667	0.267
	56.00	0.667	0.200
	57.75	0.533	0.200

BCI = bladder contractility index, Pdet.Qmax = maximum urinary flow rate during the voiding phase and the corresponding detrusor pressure.

## 4. Discussion

The main factors affecting voiding symptoms in BPH patients include BOO and bladder detrusor contractility. One possible cause of DU in patients with BOO is elevated bladder pressure during voiding, which causes temporary ischemia of the detrusor muscle. Post-voiding, detrusors may suffer from ischemia–reperfusion injury.^[5]^ Long-term ischemia–reperfusion damage can lead to pathological changes in the bladder smooth muscle and nerve cells. Additionally, increased residual urine can cause detrusor to remain in a high-load state, with bacterial metabolites in the urine providing stimulation. Along with physiological factors such as aging, these lead to detrusor damage.^[6]^ Liu Shenglai et al^[7]^ investigated the molecular mechanisms of BOO to DU. They proposed that sustained intravesical pressure due to BOO initiates a cascade of events beginning with mechanical tension stress, hypoxia, ischemia–reperfusion injury, alteration of cellular metabolism, and remodeling of the extracellular matrix, resulting in irreversible pathological changes to the detrusor muscle.

DU can be categorized into 3 stages based on the degree and duration: bladder smooth muscle hypertrophy, compensation phase, and decompensation phase. Early pathological changes in BPH involve increased bladder outlet resistance and compensatory hypertrophy of the detrusor, which enhances contractility. Prolonged BOO can lead to impaired detrusor contraction, muscle atrophy, and further detrusor damage due to bladder overdistension. As the condition progresses to decompensation, detrusor fibrosis ensues,^[8]^ and cystoscopy reveals bladder trabeculation and pseudodiverticula.

Research has shown that during the compensatory phase of detrusor function, relieving BOO can allow for recovery or partial recovery. However, once decompensation occurs, detrusor damage becomes irreversible even if BOO is relieved.^[9]^ Historically, the assessment of detrusor contractility has lacked specific diagnostic tools, relying mainly on subjective clinical judgments or estimations based on detrusor thickness via ultrasound, which are not highly accurate.^[10]^ Some researchers have combined parameters^[11]^ such as fQmax, bladder emptying rate, and duration of symptoms to formulate a risk prediction model for detrusor function, but lack stronger evidence. Currently, urodynamics is the gold standard for evaluating BOO and detrusor contractility,^[12]^ although it is not widely available in grassroots hospitals, where BPH surgeries are frequent. Traditionally, BPH patients with DU are unsuitable for surgery, as DU is a risk factor for poor postoperative outcomes.^[13]^ Poor surgical outcomes and potential litigation often lead to the long-term use of urinary catheters, severely affecting the quality of life and increasing complications, eliminating the chance for bladder recovery, and potentially causing upper urinary tract obstruction, affecting renal function, and risking life-threatening conditions.

Recent studies indicate that even patients with DU can benefit from surgery; however, there is no consensus or large-scale documentation. Some findings show that 78% of BPH patients with DU experienced significant recovery in bladder contractility 6 months postsurgery, with 94.7% being able to void independently.^[14]^ Tao et al^[15]^ found that most DU patients had effective postoperative outcomes, although when Pdet.Qmax was ≤20 cm H_2_O, only QOL scores improved, without a clear threshold being defined. Wang Dong et al^[16]^ suggested that a Pdet.Qmax of ≤32.5 cm H_2_O did not result in satisfactory improvements in objective indicators postsurgery.

In this study, we found that most BPH patients with DU achieved significant improvement by surgically relieving BOO. Improvements were noted in subjective scores, fQmax, and voided volume, although significant PVR differences were observed only in the W+ group. The extent of improvement varied significantly among different levels of detrusor contractility, aligning with the traditional belief that better bladder function predicts better surgical outcomes. Although surgical efficacy decreases with declining detrusor contractility, approximately 50% of severely impaired patients still achieve desirable results. Although some patients retain significant residual urine postoperatively, regular follow-ups have shown no upper urinary tract damage, suggesting that lower postsurgical bladder storage pressures and outlet pressures help prevent ureteral reflux. Increased PVR may be related to improved hydration habits postsurgery, measurement techniques, or examination timing, whereas VV and PVR variability may be related to factors such as postsurgical prostate cavity enlargement, leading to inconsistencies. IPSS and QOL scores are highly subjective, especially for those with urinary retention who can void independently postoperatively, offering satisfaction, even with minimal improvements in flow rates.

In comparing surgical outcomes among groups, the improvement in IPSS between the VW and W− groups showed no significant difference, nor did VV and PVR between groups, possibly related to patient satisfaction factors. These indicators, although somewhat valuable, show that both physicians and patients prioritize improvements in flow rates. Using a change in fQmax of ≥5 mL/s as an efficacy standard across all groups yielded an effective response rate of 72.4%, which was satisfactory, especially for urinary retention cases. For patients with severe DU, similar to those in the VW group, our study identifies 3 predictive factors for surgical outcomes. When the BCI exceeds 40 to 42, the accuracy (sensitivity) is high and the misdiagnosis rate (1-specificity) is low, with a Youden index of approximately 0.6, indicating high reliability. Pdet.Qmax exceeding 36.5 cm H_2_O during urodynamic testing offers a Youden index (the Youden index [J] is a statistical measure used to evaluate the performance of a diagnostic or classification test. It assesses a test’s ability to balance sensitivity [true positive rate] and specificity [true negative rate], providing a single value that summarizes its overall discriminative power. J = sensitivity + specificity − 1) of approximately 0.533, which is also reliable. A prostate volume > 55 mL yields a Youden index of approximately 0.467, which is considered moderate. Thus, we propose that in VW patients, surgery can be considered with thorough preoperative communication if the BCI is >40 to 42. Otherwise, informing patients about possibly unsatisfactory outcomes and considering less invasive options, such as catheters or bladder stomas, are advisable.

Overall, there are some limitation in our study. More data are needed to better evaluate preoperative indicators in patients with urinary retention and define effective treatment standards. In future studies, we will place greater emphasis on appropriate controls, replication, and sample sizes.

## Author contributions

**Funding acquisition:** Yuhong Xin.

**Formal analysis:** Bingkun Li.

**Investigation:** Yuhong Xin.

**Resources:** Zhenhua Zhao.

**Supervision:** Bingkun Li.

**Writing – original draft:** Yuhong Xin, Bingkun Li.

**Writing – review & editing:** Dai Li, Shuzhen Lin, Riqiang Gan, Lijuan Li, Aili Zhao, Chao Yang.
